# Synthesis and Self-Assembly Properties of Bola-Amphiphilic Glycosylated Lipopeptide-Type Supramolecular Hydrogels Showing Colour Changes Along with Gel–Sol Transition

**DOI:** 10.3390/ijms22041860

**Published:** 2021-02-13

**Authors:** Naoki Tsutsumi, Akitaka Ito, Azumi Ishigamori, Masato Ikeda, Masayuki Izumi, Rika Ochi

**Affiliations:** 1 Graduate School of Integrated Arts and Sciences, Kochi University, 2-5-1, Akebono-cho, Kochi 780-8520, Japan; b20m6g46@s.kochi-u.ac.jp (N.T.); izumi@kochi-u.ac.jp (M.I.); 2 School of Environmental Science and Engineering, Kochi University of Technology, Kami, Kochi 782-8502, Japan; ito.akitaka@kochi-tech.ac.jp; 3 Research Center for Molecular Design, Kochi University of Technology, Kami, Kochi 782-8502, Japan; 4 Faculty of Science, Kochi University, 2-5-1, Akebono-cho, Kochi 780-8520, Japan; aswositm24@eis.hokudai.ac.jp; 5 Department of Chemistry and Biomolecular Science, Faculty of Engineering, Gifu University, 1-1 Yanagido, Gifu 501-1193, Japan; m_ikeda@gifu-u.ac.jp; 6 United Graduate School of Drug Discovery and Medical Information Sciences, Gifu University, 1-1 Yanagido, Gifu 501-1193, Japan; 7 Interdisciplinary Science Unit, Multidisciplinary Sciences Cluster, Research and Education Faculty, Kochi University, 2-5-1, Akebono-cho, Kochi 780-8520, Japan; 8 Faculty of Science and Technology, Kochi University, 2-5-1, Akebono-cho, Kochi 780-8520, Japan

**Keywords:** self-assembly, peptide, supramolecular hydrogel, chromism

## Abstract

Supramolecular hydrogels formed by self-assembly of low-molecular-weight amphiphiles (hydrogelators) have attracted significant attention, as smart and soft materials. However, most of the observed stimuli-responsive behaviour of these supramolecular hydrogels are limited to gel–sol transitions. In this study, we present bola-amphiphilic glycosylated lipopeptide-type supramolecular hydrogelators that exhibit reversible thermochromism along with a gel–sol transition. The bola-amphiphiles have mono-, di-, tri- or tetra-phenylalanine (F) as a short peptide moiety. We investigate and discuss the effects of the number of F residues on the gelation ability and the morphology of the self-assembled nanostructures.

## 1. Introduction

Supramolecular hydrogels constructed through the self-assembly of low molecular-weight amphiphiles (hydrogelator) via weak non-covalent interactions, such as hydrogen bonding, hydrophobic interactions, *π*-*π* stacking, electrostatic interactions have attracted significant attention, as smart and soft materials [1,2,3,4,5,6,7,8,9,10,11,12,13]. Many supramolecular hydrogels capable of rapidly responding to external stimuli, such as chemical additives [14,15,16,17,18,19,20,21] and biological molecules [22,23,24,25,26,27,28,29,30,31,32,33], have been reported. However, most of the observed stimuli-responsive behaviour of these supramolecular hydrogels is limited to gel–sol transitions. Supramolecular hydrogels that exhibit a colour change (i.e., chromism) in response to the desired external stimuli are useful for developing practical sensing materials [34,35,36,37,38,39,40].

**Figure 1:**
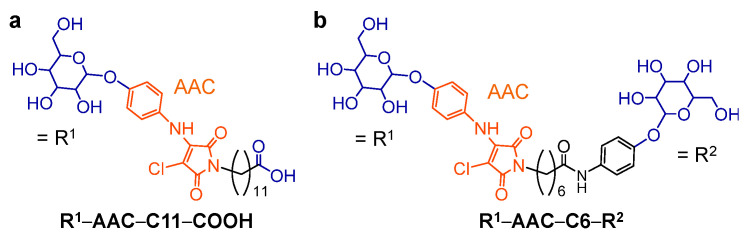
Chemical structures of bola-amphiphilic glycolipid-type hydrogelators possessing an *N*-alkyl-2-anilino-3-chloromaleimide (AAC) moiety (R^1^, R^2^ = α- or β-D-glucose (α/βGlc), α- or β-D-galactose (α/βGal); (**a**) R^1^–AAC–C11–COOH [36], (**b**) R^1^–AAC–C6–R^2^ [40].

We developed supramolecular hydrogels that exhibit thermochromism along with a gel–sol transition in which the hydrogel state was yellow and the solution state was orange (Figure 1) [36,40]. The hydrogelators have an *N*-alkyl-2-anilino-3-chloromaleimide (AAC) moiety as a chromophore, capable of acting as a probe to readout the self-assembly state. Considering its solution state counterpart, the blue-shifted absorption band in the hydrogel state indicates that the AAC moiety in the hydrogelators was stacked in an *H*-type aggregation mode [41,42]. Despite the usefulness of this system as a colorimetric assay, the molecular design of hydrogelators was limited to the glycolipid-type bola-amphiphiles with hydrophilic moiety (saccharide or carboxy group) at each end of the hydrophobic core, including the AAC moiety and the hydrocarbon chain.

In this paper, we report the design, synthesis, and self-assembly properties of glycosylated lipopeptide-type bola-amphiphiles to diversify the molecular design of our colour-change hydrogelation system. The lipopeptides are a class of molecules with one or more lipid chains attached to a short peptide. Recently, self-assembled peptide-based nanomaterials have attracted significant attention, as functional materials [43,44,45,46,47,48,49]. Short peptide-based amphiphiles have been studied as hydrogelators due to their ability to assemble into a large range of novel nanostructures and their rational design for various applications, such as molecular sensors, tissue engineering, and drug-delivery systems [50,51,52,53,54,55,56]. The molecular configurations and intramolecular interactions of short peptides can be controlled by the amino acid sequence. Aromatic amino acids, such as phenylalanine (F), tyrosine (Y), and tryptophan (W) are a popular type of building block with aromatic *π*-*π* interactions and hydrophobic interaction. Hydrogelators containing diphenylalanine (F2) peptide, which is the core motif of Alzheimer’s β-amyloid peptide or more extended aromatic sequences have been investigated [57,58,59]. F2 peptide can self-assemble into a nanostructure that is obtained by combining hydrogen bonding and *π*-*π* stacking interactions. Despite the significant attention on F2-based hydrogels, a few examples of triphenylalanine (F3)- or tetraphenylalanine (F4)-based hydrogels and self-assembled nanostructures have been investigated [60,61,62,63,64,65]. Here, we evaluate the effects of the number of F residues on the gelation ability and the morphology of the self-assembled nanostructures.

## 2. Results and Discussion

### 2.1. Molecular Design of Glycosylated Lipopeptide-type Bola-amphiphiles

**Figure 2:**
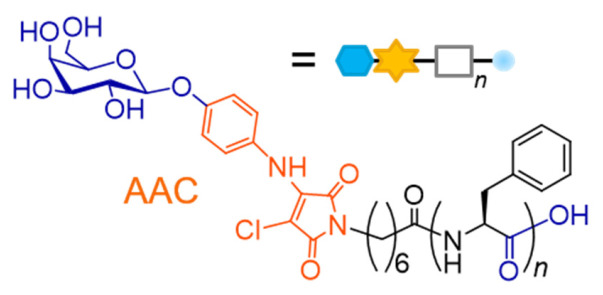
Chemical structures of glycosylated lipopeptide-type bola-amphiphiles βGal–AAC–C6–F*n* (*n* = 1–4).

**Scheme 1:**
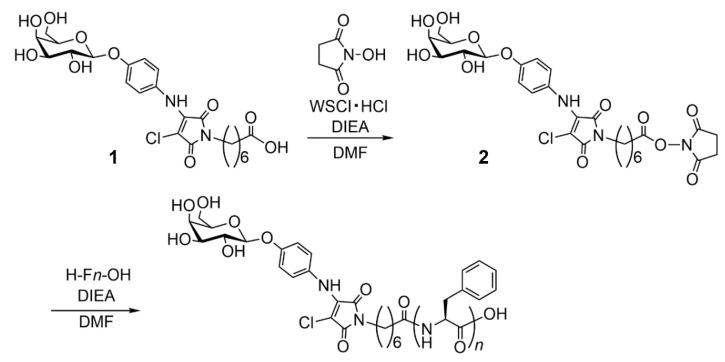
Synthesis of βGal–AAC–C6–F*n* (*n* = 1–4).

We designed and synthesized the glycosylated lipopeptide-type bola-amphiphiles with the general formula β-D-galactose (βGal)–AAC–C6–F*n* (*n* = 1–4) (Figure 2, Scheme 1). The saccharide structure and hydrocarbon chain length are essential factors for the self-assembly property of glycolipid-type amphiphiles [36,40,66,67,68,69,70,71,72]. We fixed the saccharide structure as βGal and hydrocarbon chain length as the C6 spacer based on the previous study [40]. Thus, we evaluated the effect of the number of F residues on the self-assembly properties of the prepared bola-amphiphiles.

### 2.2. Self-Assembly Properties of Glycosylated Lipopeptide-Type Bola-Amphiphiles

**Figure 3:**
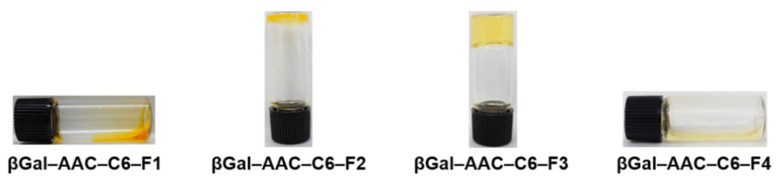
Photographs of compounds at room temperature (around 23 °C); [βGal–AAC–C6–F1] = 2.4 wt%, [βGal–AAC–C6–F2] = 2.4 wt% (CGC), [βGal–AAC–C6–F3] = 0.19 wt% (CGC), and [βGal–AAC–C6–F4] = 0.35 wt% in 200 mM HEPES–NaOH buffer (pH 8.0).

The gelation ability of the glycosylated lipopeptide-type bola-amphiphiles in 200 mM HEPES–NaOH buffer (pH 8.0) was screened using the tube-inversion method (Table 1, Figure 3). βGal–AAC–C6–F2 formed unstable partial hydrogel, and βGal–AAC–C6–F3 formed stable transparent hydrogel at their critical gelation concentrations (CGCs). However, even at sufficiently high concentration (about 10 wt%), βGal–AAC–C6–F1 remained in solution state (Figure 3a). This behaviour may be attributed to the higher water solubility compared to the other compounds. Furthermore, the sample of βGal–AAC–C6–F4, which showed the lowest solubility, was dispersed at 0.35 wt%. These results indicate that differences in the combination of intermolecular hydrogen bonding between amide groups and side-chain *π*-*π* stacking interactions, which were caused by the difference in the number of F residues, have a significant effect on the self-assembly properties. It was revealed that βGal–AAC–C6–F3 has the optimal assembly properties for gel formation. We obtained that the compound’s maximum absorption wavelength (*λ*_max_) in 200 mM HEPES–NaOH buffer (pH 8.0) shifted to shorter wavelength as the number of F increased (Table 1, Figure S1, ESI† for absorption spectra), suggesting the larger intermolecular interaction of the molecule. The difference in the chemical structure is only the number of F unit, however, the abrupt change in the phase behaviour was observed. It is presumed that the differences in the aromatic *π*–*π* interactions derived from the side chain phenyl groups of F and the hydrogen bonding mode derived from the difference in the amide bonds greatly contribute to the self-assembly ability of the molecules.

**Figure 4:**
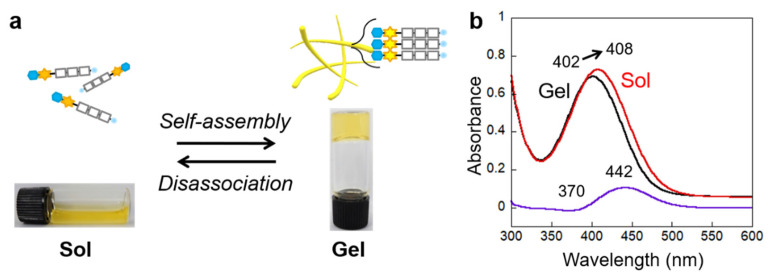
(**a**) Photographs and schematics of a supramolecular hydrogel of βGal–AAC–C6–F3 exhibiting reversible thermal gel–sol transition and thermochromism and (**b**) UV–Vis absorption spectral changes of the supramolecular hydrogel of βGal–AAC–C6–F3 upon heating (black line: 25 °C (gel state), red line: 85 °C (solution state), purple line: differential spectrum). Conditions: [βGal–AAC–C6–F3] = 0.19 wt%, 200 mM HEPES–NaOH buffer (pH 8.0).

The above gelation process was thermally reversible, as shown in Figure 4a for βGal–AAC–C6–F3 (*T*_gel_: 78 °C at 0.19 wt% (CGC)), and a chromic change upon the sol–gel transition was observed similarly to the existing system [39]. The UV–Vis absorption spectral analysis confirmed the colour change. The absorption band arising from the AAC moiety was bathochromically shifted from the absorption maximum being 402 nm for the hydrogel state at 25 °C to 408 nm for the solution state at 85 °C (Figure 4b and Figure S2, ESI† for temperature-dependent absorption spectral change). The differential spectrum showed an increase in absorbance at 442 nm and a decrease of 370 nm. Considering its solution state counterpart, the blue-shifted absorption band in the hydrogel state (Δ*λ*_max_ = 6 nm) indicates that the AAC moieties in the hydrogel are stacked in an *H*-type aggregation mode. However, the visual colour change between the gel state and the solution state of βGal–AAC–C6–F3 sample was scarce (Figure 4a) compared to previously reported glycolipid-type hydrogels [35,39]. The cause of this phenomenon is unknown, and we intend to investigate the cause in the future.

### 2.3. Morphology of the Self-Assembled Glycosylated Lipopeptide-Type Bola-Amphiphiles

**Figure 5:**
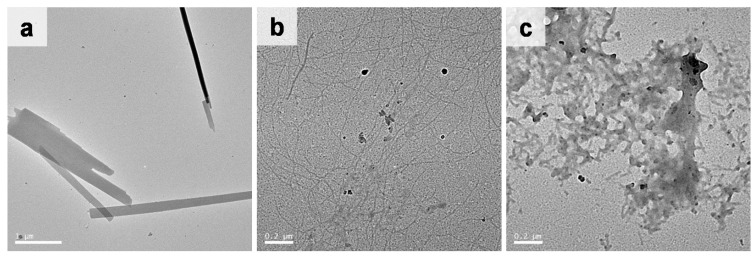
Representative TEM images of (**a**) βGal–AAC–C6–F2, (**b**) βGal–AAC–C6–F3, and (**c**) βGal–AAC–C6–F4 samples after being transferred onto elastic carbon-coated grids and dried in vacuo. Conditions: [βGal–AAC–C6–F2] = 2.4 wt% (CGC), [βGal–AAC–C6–F3] = 0.19 wt% (CGC), and [βGal–AAC–C6–F4] = 0.35 wt%, 200 mM HEPES–NaOH buffer (pH 8.0).

Insight into the morphology of the self-assembled structures was obtained by transmission electron microscopy (TEM) on the βGal–AAC–C6–F2 and βGal–AAC–C6–F3 hydrogels, and βGal–AAC–C6–F4 dispersion. TEM images showed that βGal–AAC–C6–F2 self-assembled into nanoribbons with an averaged width of several tens of nm and up to ca. 100 nm and length of several micrometres (Figure 5a). On the other hand, βGal–AAC–C6–F3 self-assembled into longer and thinner one-dimensional (1D) nanofibers with an averaged width of ca. 25 nm, thereby facilitating the formation of a highly entangled three-dimensional (3D) network (Figure 5b). These conventional TEM images are not cryo-TEM and thus there is the potential influence of the sample preparation process (especially during the drying process) on the observed morphologies. Nevertheless, we speculate that the longer and thinner nanofibers of βGal–AAC–C6–F3, instead of the straight nanoribbons of βGal–AAC–C6–F2, should be responsible for the stable hydrogel formation of βGal–AAC–C6–F3 even at a lower concentration. The detail in the molecular assembly mode and the difference is under investigation. In contrast, the self-assembled structures of βGal–AAC–C6–F4 dispersion (non-hydrogel) were non-networked aggregates of shorter nanofibers (Figure 5c).

## 3. Materials and Methods

### 3.1. Generals

Chemical reagents were purchased from Tokyo Chemical Industry Co., Ltd., FUJIFILM Wako Pure Chemical Corporation, Watanabe Chemical Industries, Ltd. and Bachem Holding AG., and used without further purification. Thin-layer chromatography (TLC) was performed on TLC silica gel 60F_254_ (Merck). Column chromatography was performed on silica gel 60N (Kanto Chemical Co., Inc., spherical neutral, 63 to 210 µm). ^1^H and ^13^C NMR spectra were recorded on a JEOL ECA500 spectrometer in CDCl_3_, CD_3_OD or dimethyl sulfoxide-*d*_6_ (DMSO-*d*_6_) with tetramethylsilane (TMS) or residual non-deuterated solvents as the internal references. Multiplicities are abbreviated as follows: s = singlet, d = doublet, t = triplet, q = quartet, m = multiplet, dd = double doublet, and br = broad. LRMS (ESI-MS) analysis was conducted using a Bruker amaZon SL mass spectrometer. HRMS (ESI-FT-ICR-MS) analyses were conducted using a Bruker Solarix spectrometer. The absorption spectra were measured using a Jasco V-650 spectrometer equipped with an ETCS-761 temperature controller. FT-IR spectra were recorded on a JEOL FT/IR-4100 spectrometer using KBr pellets in the range of 4000 to 400 cm^−1^.

### 3.2. Gelation Test

The compounds (typically, 2.0 mg) were suspended in 200 mM HEPES–NaOH buffer (pH 8.0) in a mighty vial. The suspensions were heated to form homogeneous solutions. Then, the hot solution was cooled to room temperature (around 23 °C) and incubated for 10 min. Gelation was confirmed by the gravitational flux with inversion of the vial. When no fluid ran down the wall of the test tube upon inversion of the vial, we judged it to be gel. Upon confirming the gel formation, the buffer was added to the samples, heated to dissolution and cooled to room temperature. This process was repeated until the gel formation could no longer be observed. The CGC was considered as the last concentration at which a stable gel phase could be observed.

### 3.3. Measurement of the Gel–Sol Transition Temperature (T_gel_)

The gel–sol phase transition behaviour of the βGal–AAC–C6–F3 hydrogel (0.19 wt% (CGC) in 200 mM HEPES–NaOH buffer (pH 8.0)) was determined using the vial inversion method. The inverted gel in the vial was placed in an oil bath, which was heated from 25 °C to 85 °C, at a rate of 1 °C/step. The vial was immersed at each temperature for 1 min to equilibrate. The temperature, at which the sample completely dissolved, was defined as the *T*_gel_ value of the gel.

### 3.4. Measurements of the Absorption Spectra of the Compounds

An aqueous suspension of the compounds in 200 mM HEPES–NaOH buffer (pH 8.0) was heated to form a homogeneous solution. The hot solution was transferred into a quartz cell (path length: 0.1 mm (assembled quartz cell, GL Sciences Inc., cat. no. AB10-UV-0.1 with cell adaptor, GL Sciences Inc., cat. no. CAS-10-1) for βGal–AAC–C6–F1 and βGal–AAC–C6–F2, or 1 mm for βGal–AAC–C6–F3 and βGal–AAC–C6–F4) and stored at room temperature for 10 min. The absorption spectra were measured at room temperature. Conditions: [βGal–AAC–C6–F1] = 2.4 wt%, [βGal–AAC–C6–F2] = 2.4 wt% (CGC), [βGal–AAC–C6–F3] = 0.19 wt% (CGC), and [βGal–AAC–C6–F4] = 0.35 wt%, 200 mM HEPES–NaOH buffer (pH 8.0).

### 3.5. Measurements of the Temperature-Dependent Absorption Spectral Changes of βGal–AAC–C6–F3 Hydrogel

An aqueous suspension of βGal–AAC–C6–F3 (0.19 wt% (CGC) in 200 mM HEPES–NaOH buffer (pH 8.0)) was heated to form a homogeneous solution. The hot solution (400 mL) was transferred into a quartz cell (path length: 1 mm) and stored at room temperature for 10 min to complete the gelation. The absorption spectra were measured upon heating from 25 to 85 °C.

### 3.6. TEM Observation

Sample (ca. 10 µL) was dropped on a copper TEM grid covered by an elastic carbon-support film (20 to 25 nm) with a filter paper underneath. Then, the excess solution was blotted with the filter paper immediately. The TEM images were acquired using a JEOL JEM-2100F (accelerating voltage: 200 kV) equipped with a CCD camera. Conditions: [βGal–AAC–C6–F2] = 2.4 wt% (CGC), [βGal–AAC–C6–F3] = 0.19 wt% (CGC), and [βGal–AAC–C6–F4] = 0.35 wt%, 200 mM HEPES–NaOH buffer (pH 8.0).

### 3.7. Synthesis

Compound **1** was synthesized according to previously reported methods [39]. H-FF-OH was purchased from Bachem Holding AG. H-FFF-OH and H-FFFF-OH were synthesized by standard liquid phase synthesis using *N*-α-(9-Fluorenylmethoxycarbonyl)-L-phenylalanine (Fmoc-F-OH) and L-phenylalanine *t*-butyl ester hydrochloride (H-F-OBu*^t^*•HCl) [73]. All the amino acids and Fmoc protected peptides were conjugated via active ester method by using 1-[1-(cyano-2-ethoxy-2-oxoethylideneaminooxy)-dimethylamino-morpholino]-uronium hexafluorophosphate (COMU) in the presence of *N*,*N*-diisopropylethylamine (DIEA) in dry *N*,*N*-dimethylformamide (DMF) under an N_2_ atmosphere. Fmoc deprotection was done with 20% piperidine in DMF. *t*-Butyl ester deprotection was done with trifluoroacetic acid:H_2_O = 95:5 (*v*/*v*).

#### 3.7.1. Synthesis of Compound **2**

After cooling in an ice bath, *N*-hydroxysuccinimide (52 mg, 0.45 mmol, 1.5 eq.) and water soluble carbodiimide hydrochloride (WSCI•HCl, 116 mg, 0.60 mmol, 2.0 eq.) were added to a solution of **1** (160 mg, 0.30 mmol, 1.0 eq.) in dry DMF (6 mL), and the mixture was stirred at room temperature overnight under an N_2_ atmosphere. The solvent was then evaporated, and residue was purified by column chromatography (SiO_2_, CH_2_Cl_2_:MeOH = 6:1 (*v*/*v*)). Then, the residue was further purified by reprecipitation with diethyl ether (Et_2_O) for two times. The resulting product was dried under vacuum to give compound **2** (113 mg, 59%) as a yellow powder. ^1^H NMR (500 MHz, CD_3_OD): *δ* (ppm) = 1.31–1.37 (m, 3H), 1.43–1.49 (m, 3H), 1.58–1.64 (m, 3H), 1.69–1.75 (m, 3H), 2.62 (q, *J* = 7.1 Hz, 3H), 2.80 (q, *J* = 6.6 Hz, 4H), 3.51 (t, 2H), 3.57 (dd, *J_1_* = 3.4 Hz, *J_2_* = 9.7 Hz, 1H), 3.67 (q, *J* = 5.7 Hz, 1H), 3.72–3.80 (m, 3H), 3.89 (d, *J* = 3.5 Hz, 1H), 4.89 (d, *J* = 7.4 Hz, 1H), and 7.09–7.13 (m, 4H). ^13^C NMR (125 MHz, CD_3_OD): *δ* (ppm) = 25.51, 26.48, 27.20, 29.15, 29.35, 31.45, 39.07, 62.41, 70.19, 72.22, 74,.75, 76.88, 92.05, 103.07, 117.64, 126.70, 132.29, 139.69, 157.19, 167.07, 169.76, 170.26, 171.90. LRMS (ESI-TOF, positive mode): Calcd. for [M(C_27_H_32_ClN_3_O_12_)+Na]^+^: *m/z* = 648.2; Found: 648.2.

#### 3.7.2. Synthesis of βGal–AAC–C6–F1

H-F-OH (5.8 mg, 0.035 mmol, 1.1 eq.) and DIEA (6.4 µL, 0.038 mmol, 1.2 eq.) were added to a solution of **2** (20 mg, 0.032 mmol, 1.0 eq.) in dry DMF (2 mL), and the mixture was stirred at room temperature overnight under an N_2_ atmosphere. The solvent was then evaporated, and residue was purified by column chromatography (SiO_2_, CH_2_Cl_2_:MeOH = 2:1 (*v*/*v*)). Then, the residue was further purified by reprecipitation with Et_2_O for two times. The resulting product was dried under vacuum to give βGal–AAC–C6–F1 (17 mg, 78%) as a yellow powder. ^1^H NMR (500 MHz, CD_3_OD): *δ* (ppm) = 1.16–1.28 (m, 4H), 1.43–1.55 (m, 4H), 2.09–2.14 (m, 2H), 2.88–3.00 (m, 2H), 3.47 (t, *J_1_* = 7.2 Hz, 2H), 3.57 (dd, *J_1_* = 2.9 Hz, *J_2_* = 9.8 Hz, 1H), 3.66–3.70 (m, 1H), 3.71–3.80 (m, 3H), 3.88 (d, *J* = 3.5 Hz, 1H), 4.52 (q, *J* = 4.7 Hz, 1H), 4.58 (s, 1H), 7.09–7.15 (m, 3H), 7.18–7.20 (m, 3H), and 7.33–7.24 (m, 3H). ^13^C NMR (125 MHz, DMSO-*d*_6_): *δ* (ppm) = 25.17, 26.05, 28.05, 32.77, 33.05, 35.20, 36.98, 37.84, 48.70, 53.71, 60.48, 68.21, 70.39, 73.57, 90.00, 101.26, 115.89, 125.61, 126.33, 128.14, 129.30, 138.16, 147.53, 151.05, 155.31, 165.55, 167.55, 172.12, 173.84. HRMS (ESI-FT-ICR, positive mode): Calcd. for [M(C_32_H_38_ClN_3_O_11_)+H]^+^: *m/z =* 676.2268; Found: 676.2258. FT-IR (KBr pellet): *v* = 3422.1, 3029.6, 2937.1, 2861.8, 2359.5, 2344.1, 1771.3, 1712.5, 1654.6, 1607.4, 1508.1, 1442.5, 1409.7, 1231.3, 1151.3, 1074.2, 834.1, 743.4, 703.9 cm^–1^.

#### 3.7.3. Synthesis of βGal–AAC–C6–F2

H-FF-OH (11 mg, 0.035 mmol, 1.1 eq.) and DIEA (6.4 µL, 0.038 mmol, 1.2 eq.) were added to a solution of **2** (20 mg, 0.032 mmol, 1.0 eq.) in dry DMF (4 mL), and the mixture was stirred at room temperature overnight under an N_2_ atmosphere. The solvent was then evaporated, and residue was purified by column chromatography (SiO_2_, CH_2_Cl_2_:MeOH = 3:1 to 2:1 to 1:1 to 0:1 (*v*/*v*)). Then, the residue was further purified by reprecipitation with Et_2_O for two times. The resulting product was dried under vacuum to give βGal–AAC–C6–F2 (22 mg, 84%) as a yellow powder. ^1^H NMR (500 MHz, CD_3_OD): *δ* (ppm) = 1.08 (q, *J* = 8.0 Hz, 2H), 1.16 (q, *J* = 7.1 Hz, 2H), 1.34–1.38 (m, 2H), 1.48 (q, *J* = 7.4 Hz, 2H), 2.06 (t, *J* = 6.9 Hz, 1H), 2.72 (dd, *J_1_* = 5.3 Hz, *J_2_* = 13.5 Hz, 2H), 3.01 (dd, *J_1_* = 3.3 Hz, *J_2_* = 14.0 Hz, 1H), 3.13–3.17 (m, 2H), 3.43–3.48 (m, 2H), 3.57 (dd, *J_1_* = 3.2 Hz, *J_2_* = 10.0 Hz, 1H), 3.67–3.69 (m, 1H), 3.72–3.80 (m, 3H), 3.89 (d, *J* = 3.4 Hz, 1H), 4.44 (t, *J* = 5.8 Hz, 1H), 4.57–4.60 (m, 3H), 7.09–7.14 (m, 5H), and 7.15–7.27 (m, 9H). ^13^C NMR (125 MHz, DMSO-*d*_6_): *δ* (ppm) = 25.09, 25.24, 25.98, 27.97, 29.53, 33.05, 35.14, 36.92, 37.35, 48.30, 54.25, 84. 63, 93.18, 98.30, 101.26, 115.69, 125.51, 126.13, 127.94, 129.14, 129.39, 138.20, 141.81, 150.62, 151.05, 157.12, 167.39, 169.91, 170.97, 171.35, 172.82. HRMS (ESI-FT-ICR, positive mode): Calcd. for [M(C_41_H_47_ClN_4_O_12_)+H]^+^: *m/z =* 823.2952; Found: 823.2958. FT-IR (KBr pellet): *v* = 3398.0, 3060.5, 3027.7, 2933.2, 2859.9, 2360.4, 2342.1, 1770.3, 1712.5, 1654.6, 1603.5, 1508.1, 1442.5, 1409.7, 1231.3, 1150.3, 1077.1, 832.1, 744.4, 701.0 cm^–1^.

#### 3.7.4. Synthesis of βGal–AAC–C6–F3

H-FFF-OH (16 mg, 0.035 mmol, 1.1 eq.) and DIEA (6.4 µL, 0.038 mmol, 1.2 eq.) were added to a solution of **2** (20 mg, 0.032 mmol, 1.0 eq.) in dry DMF (3 mL), and the mixture was stirred at 50 °C overnight under an N_2_ atmosphere. The solvent was then evaporated, and residue was purified by column chromatography (SiO_2_, CH_2_Cl_2_:MeOH = 2:1 to 1:1 to 0:1 (*v*/*v*)). Then, the residue was further purified by reprecipitation with Et_2_O for two times. The resulting product was dried under vacuum to give βGal–AAC–C6–F3 (18 mg, 58%) as a yellow powder. ^1^H NMR (500 MHz, CD_3_OD): *δ* (ppm) = 1.05–1.10 (m, 2H), 1.16 (q, *J* = 7.2 Hz, 2H), 1.32–1.39 (m, 2H), 1.48 (q, *J* = 7.1 Hz, 2H), 2.01 (t, *J* = 7.7 Hz, 2H), 2.79–2.85 (m, 1H), 2.99–3.05 (m, 2H), 3.12–3.16 (m, 2H), 3.18–3.21 (m, 1H), 3.43–3.49 (m, 3H), 3.57 (dd, *J*_1_ = 3.7 Hz, *J*_2_ = 10.0 Hz, 1H), 3.68 (t, *J* = 5.7 Hz, 1H), 3.72–3.80 (m, 3H), 3.89 (d, *J* = 4.0 Hz, 1H), 4.44–4.47 (m, 1H), 4.56–4.61 (m, 3H), 7.11–7.13 (m, 5H), and 7.15–7.27 (m, 14H). HRMS (ESI-FT-ICR, positive mode): Calcd. for [M(C_50_H_56_ClN_5_O_13_)+H]^+^: *m/z =* 970.3636; Found: 970.3606. FT-IR (KBr pellet): *v* = 3403.7, 3083.6, 3062.4, 3027.7, 2933.2, 2859.0, 2360.4, 2342.1, 1772.3, 1708.6, 1653.7, 1539.9, 1523.5, 1508.1, 1455.0, 1440.6, 1409.7, 1303.6, 1230.4, 1151.3, 1078.0, 817.7, 744.4, 699.1, 669.2 cm^–1^.

#### 3.7.5. Synthesis of βGal–AAC–C6–F4

H-FFFF-OH (13 mg, 0.018 mmol, 1.1 eq.) and DIEA (4.0 µL, 0.024 mmol, 1.5 eq.) were added to a solution of **2** (10 mg, 0.016 mmol, 1.0 eq.) in dry DMF (5 mL), and the mixture was stirred at 50 °C overnight under an N_2_ atmosphere. The solvent was then evaporated, and residue was purified by column chromatography (SiO_2_, CH_2_Cl_2_:MeOH = 2:1 to 1:1 to 0:1 (*v*/*v*)). Then, the residue was further purified by reprecipitation with Et_2_O for two times. The resulting product was dried under vacuum to give compound βGal–AAC–C6–F4 (12 mg, 66%) as a yellow powder. ^1^H NMR (500 MHz, DMSO-*d*_6_): *δ* (ppm) = 0.81–0.86 (m, 1H), 0.96–1.01 (m, 1H), 1.07 (q, *J* = 7.6 Hz, 2H), 1.20–1.27 (m, 4H), 1.39 (q, *J* = 6.0 Hz, 1H), 1.76 (s, 1H), 1.89 (q, *J* = 6.9 Hz, 1H), 2.47–2.51 (m, 2H), 2.55 (m, *J* = 6.6 Hz, 2H), 2.59 (m, *J* = 13.7 Hz, 1H), 2.69–2.76 (m, 2H), 2.81–2.85 (m,1H), 2.72 (dd, *J_1_* = 5.2 Hz, *J_2_* = 13.2 Hz, 2H), 3.01–3.07 (m, 4H), 3.46 (t, *J* = 4.9 Hz, 1H), 3.50–3.54 (m, 3H), 3.66 (t, *J* = 3.4 Hz, 1H), 3.91 (d, *J* = 6.9 Hz, 1H), 4.34–4.39 (m, 2H), 4.43–4.48 (m, 2H), 4.69–4.71 (m, 1H), 4.76 (d, *J* = 8.0 Hz, 1H), 4.89 (d, *J* = 6.3 Hz, 1H), 5.18 (d, *J* = 5.1 Hz, 1H), 6.94–7.51 (m, 20H). 7.51 (d, *J* = 5.2. Hz, 1H), 7.81 (d, *J* = 6.9 Hz, 1H), 7.91 (d, *J* = 8.5 Hz, 1H), 8.36 (d, *J* = 6.9 Hz, 1H). HRMS (ESI-FT-ICR, positive mode): Calcd. for [M(C_59_H_65_ClN_6_O_14_)+H]^+^: *m/z =* 1117.4320; Found: 1117.4341. FT-IR (KBr pellet): *v* = 3410.5, 3282.3, 3087.5, 3066.3, 3029.6, 2931.3, 2859.0, 2360.4, 2342.1, 1771.3, 1713.4, 1639.2, 1540.9, 1524.5, 1454.1, 1441.5, 1409.7, 1229.4, 1152.3, 1077.1, 832.1, 744.4, 699.1 cm^–1^.

## 4. Conclusions

In summary, we have successfully developed glycosylated lipopeptide-type supramolecular hydrogelators exhibiting small but perceptible reversible thermochromism along with the gel–sol transition. The gelation ability and the morphology of the self-assembled nanostructures depend on the number of F residues. βGal–AAC–C6–F2 formed unstable partial hydrogel (CGC = 2.4 wt%), and βGal–AAC–C6–F3 formed stable transparent hydrogel (CGC = 0.19 wt%). On the other hand, βGal–AAC–C6–F1 and βGal–AAC–C6–F4 did not form a hydrogel. The morphology of the self-assembled nanostructures was affected by the number of F residues and in the present molecular scaffold (i.e., βGal–AAC–C6) with βGal as the saccharide structure and C6 alkyl chain as the spacer, F3 peptide was optimal for hydrogel formation. Further research into potential bio-applications, such as the development of sensing materials for peptidase, is in progress.

## Figures and Tables

**Table 1:** Gelation ability, critical gelation concentration (CGC), the gel-to-sol phase transition temperature (*T*_gel_) of hydrogels at CGC, and absorption maxima (*λ*_max_) at room temperature (around 23 °C) of lipopeptide-type bola-amphiphiles. Conditions: 200 mM HEPES–NaOH buffer (pH 8.0).

Compound	Gelation Ability	CGC [wt%]	CGC [mM]	*T*_gel_ [°C]	*λ*_max_ [nm] ^1^
βGal–AAC–C6–F1	Solution	—	—	—	416
βGal–AAC–C6–F2	Partial Gel	2.4	30	—	409
βGal–AAC–C6–F3	Transparent Gel	0.19	2.0	78	402
βGal–AAC–C6–F4	Dispersion	—	—	—	397

^1^ [βGal–AAC–C6–F1] = 2.4 wt%, [βGal–AAC–C6–F2] = 2.4 wt% (CGC), [βGal–AAC–C6–F3] = 0.19 wt% (CGC), and [βGal–AAC–C6–F4] = 0.35 wt%.

## Data Availability

The data that support the findings of this study are available from the corresponding author upon reasonable request.

## Supplementary Materials

Supplementary Materials can be found at https://www.mdpi.com/1422-0067/22/4/1860/s1.
