# Supplementary material for: Synthesis and Self-Assembly Properties of Bola-Amphiphilic Glycosylated Lipopeptide-Type Supramolecular Hydrogels Showing Colour Changes Along with Gel–Sol Transition
Source: Int J Mol Sci. 2021 Feb 13;22(4):1860. doi: 10.3390/ijms22041860 (PMC7917936; doi:10.3390/ijms22041860)
Supplement: Supplementary file 1 [file ijms-22-01860-s001.pdf]

Electronic Supplementary Information

Synthesis and Self-Assembly Properties of Bola-amphiphilic Glycosylated Lipopeptide-Type Supramolecular Hydrogels Showing Colour Changes Along with Gel–Sol Transition

Naoki Tsutsumi [1], Akitaka Ito [2,3], Azumi Ishigamori [4], Masato Ikeda [5,6], Masayuki Izumi [1,4,7,8] and Rika Ochi [1,4,7,8,*]

[1] Graduate School of Integrated Arts and Sciences, Kochi University, 2-5-1, Akebono-cho, Kochi 780-8520, Japan.
[2] School of Environmental Science and Engineering, Kochi University of Technology, Kami, Kochi 782-8502, Japan.
[3] Research Center for Molecular Design, Kochi University of Technology, Kami, Kochi 782-8502, Japan.
[4] Faculty of Science, Kochi University, 2-5-1, Akebono-cho, Kochi 780-8520, Japan; E-mail: ochi@kochi-u.ac.jp
[5] Department of Chemistry and Biomolecular Science, Faculty of Engineering, Gifu University, 1-1 Yanagido, Gifu 501-1193, Japan.
[6] United Graduate School of Drug Discovery and Medical Information Sciences, Gifu University, 1-1 Yanagido, Gifu 501-1193, Japan.
[7] Interdisciplinary Science Unit, Multidisciplinary Sciences Cluster, Research and Education Faculty, Kochi University, 2-5-1, Akebono-cho, Kochi 780-8520, Japan.
[8] Faculty of Science and Technology, Kochi University, 2-5-1, Akebono-cho, Kochi 780-8520, Japan.
* Correspondence: ochi@kochi-u.ac.jp

1. Absorption spectra of the compounds.

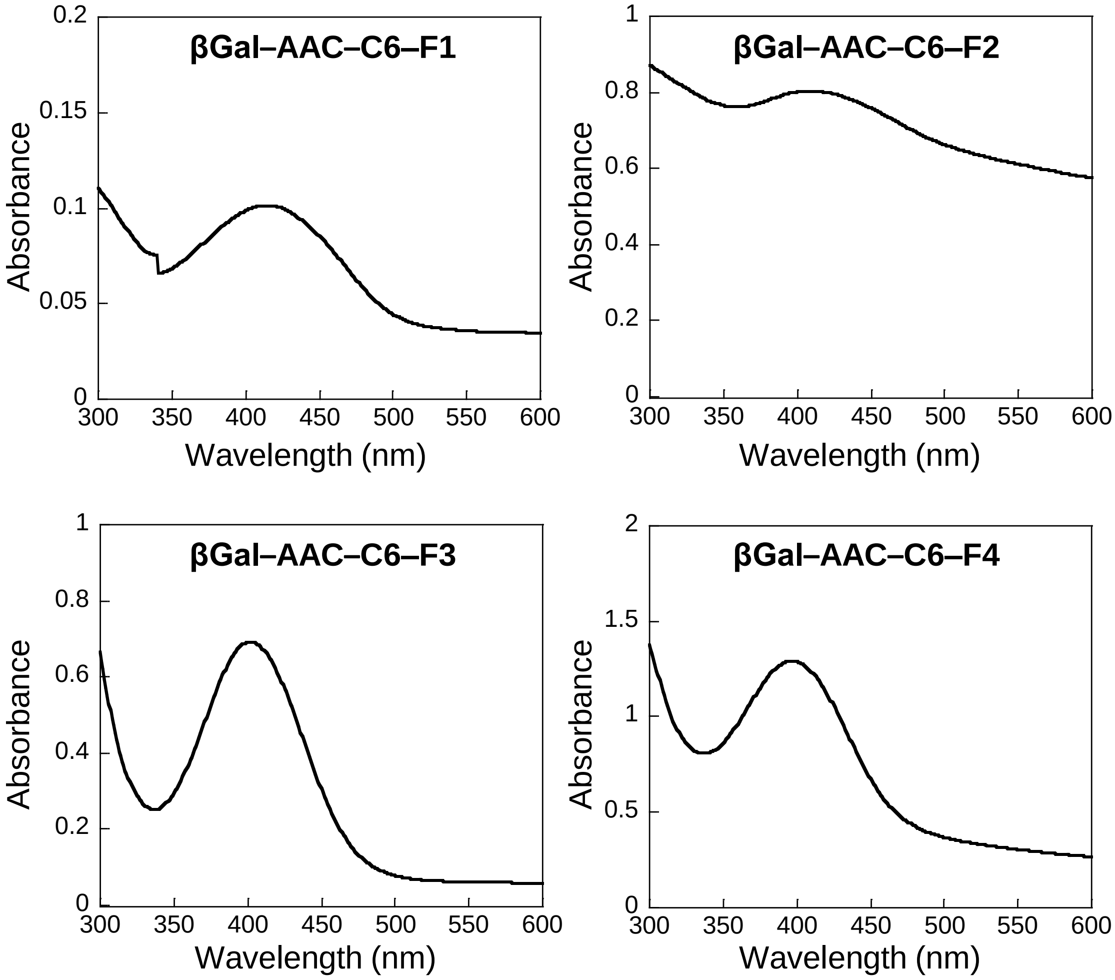

Figure S1. Absorption spectra of the compounds in 200 mM HEPES–NaOH buffer (pH 8.0) at room temperature. Conditions: [βGal–AAC–C6–F1] = 2.4 wt%, [βGal–AAC–C6–F2] = 2.4 wt% (CGC), [βGal–AAC–C6–F3] = 0.19 wt% (CGC), and [βGal–AAC–C6–F4] = 0.35 wt%. The broadening of the peak in βGal–AAC–C6–F2 would be due to non-homogeneity in the partial gel.

2. Temperature-dependent absorption spectral change of the βGal–AAC–C6–F3 hydrogel.

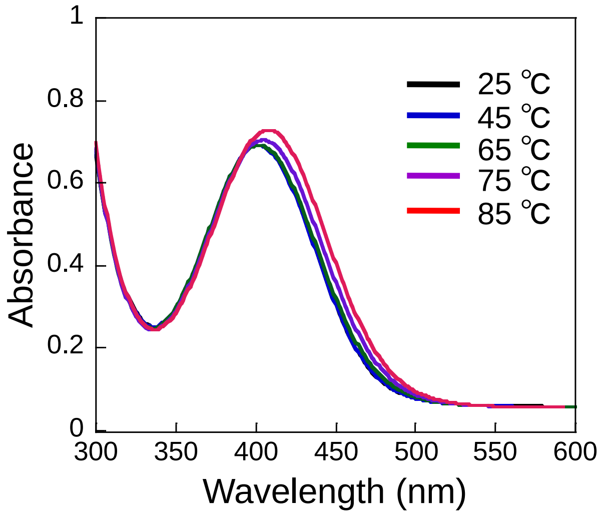

Figure S2. Absorption spectral changes of the βGal–AAC–C6–F3 hydrogel upon heating. Conditions: [βGal–AAC–C6–F3] = 0.19 wt% (CGC), 200 mM HEPES–NaOH buffer (pH 8.0).
